# Bias Evaluation of the Accuracy of Two Extraoral Scanners and an Intraoral Scanner Based on ADA Standards

**DOI:** 10.1155/2021/5535403

**Published:** 2021-06-10

**Authors:** Naiyu Cui, Jiayin Wang, Xingyu Hou, Shixun Sun, Qixuan Huang, Ho-Kyung Lim, HongXin Cai, Qi Jia, Eui-Seok Lee, Heng Bo Jiang

**Affiliations:** ^1^The Conversationalist Club, School of Stomatology, Shandong First Medical University & Shandong Academy of Medical Sciences, Tai'an, Shandong 271016, China; ^2^Department of Oral and Maxillofacial Surgery, Graduate School of Clinical Dentistry, Korea University, Seoul 02841, Republic of Korea

## Abstract

The spread and application of computer-aided design/computer-aided manufacturing (CAD/CAM) technology have contributed to the rapid development of digitalization in dentistry. The accuracy of scan results is closely related to the devising subsequent treatment plans and outcomes. Professional standards for evaluating scanners are specified in the American National Standard/American Dental Association Standard 132 (ANSI/ADA No. 132). The aims of this study were to use the three samples mentioned in ANSI/ADA No. 132 and evaluate the accuracy and reproducibility of two extraoral scanners and an intraoral scanner based on the inspection standards recommended by ANSI/ADA No. 132. In this study, two trained operators used two extraoral scanners (E4, 3Shape, Denmark & SHINING DS100+, Shining, China) and an intraoral scanner (TRIOS SERIES3, 3Shape, Denmark) to perform 30 scans of each of the three samples at a temperature of 25 ± 2°C and export standard tessellation language files and used reverse engineering software to perform measurements and iterative nearest point matching experiments. The measured values obtained were compared with the reference values measured by a coordinate measuring machine (NC8107, Leader Metrology, USA). We performed a normal distribution test (Shapiro-Wilk test), the nonparametric Kruskal-Wallis test, and an independent-samples *t*-test to analyze the reproducibility of each scan for different models. The experimental results indicate that the trueness and precision of the two extraoral scanners and the intraoral scanner had a slight mean deviation. The trueness and precision of the three scanners on the curved surface and groove areas are poor. The accuracy and reproducibility of E4 outperformed SHINING and TRIOS. The iterative closest point matching experiment also showed good matching results. The two extraoral scanners and the intraoral scanner in this study can meet the basic clinical requirements in terms of accuracy, and we hope that digital technology will be more widely used in dentistry in the future.

## 1. Introduction

In dentistry, the use of digital methods such as computer-aided design/computer-aided manufacturing (CAD/CAM) is rapidly increasing [1–4]. It improves diagnostic ability and facilitates contact between doctors and patients by allowing the rapid acquisition of 3D diagnostic information and transmission of digital data, and along with the rapid development of CAD/CAM technology in the dental field, their use in clinical diagnosis and prosthodontics is becoming more widespread [5–15]. Studies have shown that intraoral scanners are more comfortable for the patient and reduce processing time, and their accuracy and precision are within clinically acceptable limits [10, 11, 16–24]. With the progress of digital technology in the dental field, the accuracy rating of oral scanners has become increasingly important. Extraoral scanners still have higher accuracy than intraoral scanners, and the trueness and precision of intraoral scanners have become an issue of concern.

The current study shows that differences in scanning systems and methods affect experimental results to an uncertain extent [25]. Oh et al. found that different scanning strategies affect the accuracy of the results [26, 27], and a study by Giménez et al. found that operator proficiency also affects the accuracy of digital impression testing [28]. Furthermore, the accuracy of intraoral scanners changes with the length and distribution of the dental arch: the larger the scan area, the lower the accuracy with the maximum deviation of the scan in the posterior part of the dental arch [29]. The impact of external factors on the results of oral scanners has been the focus of attention, but the objective of evaluating the accuracy of oral scanners has been neglected. Therefore, it is important to objectively grade oral scanners using a more consistent scanning strategy for a standard sample.

In this study, we designed samples based on the American National Standard/American Dental Association Standard 132 (ANSI/ADA NO.132) [30] and measured the height of the crown and the radius of its top circle, the height of the inlay, and the radius of the top circle, and the distance between the reference points of the sphere. The aim of this study was to evaluate the accuracy of two extraoral scanners and an intraoral scanner by analyzing and comparing various data based on the inspection standards recommended by ANSI/ADA No. 132. Furthermore, we introduced CAD/CAM technology because it is a proven technique for manufacturing samples and computer-designed stereo images based on absolute reference measurements in the form of a coordinate measuring machine (CMM) to perform iterative closest point matching experiments to further evaluate their accuracy [31]. Meanwhile, we conducted experiments to evaluate the reproducibility of the two extraoral scanners and an intraoral scanner.

## 2. Materials and Methods

### 2.1. Fabrication of Models

**Figure 1:**
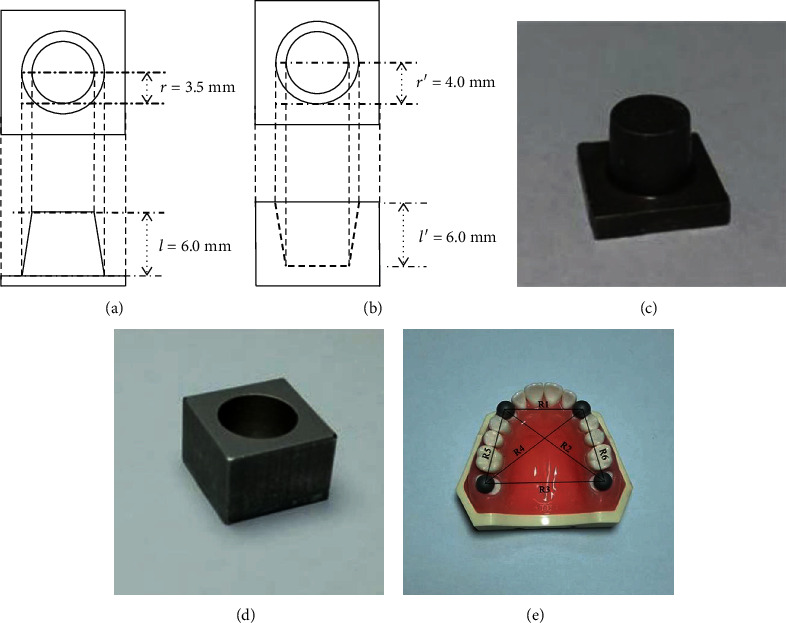
The sample observation drawn by CAD software. Top and side view of (a) sample 1 and (b) sample 2 which alphabetized the index. The manufactured (c) sample 1, (d) sample 2, and (e) sample 3 coated with alloy mentioned in ANSI/ADA No. 132. ^∗^“*r*” and “*r*′” are the radius of the circle on the top surface of samples 1 and 2. “*l*” and “*l*′” are the distance from the top to the bottom of samples 1 and 2. R1 is the distance from the center of the upper left sphere to the right. R2 is the distance from the center of the upper left sphere to the right. R3 is the distance from the center of the lower left sphere to the right. R4 is the distance from the center of the lower left sphere to the right. R5 is the distance from the center of the lower left sphere to the left. R6 is the distance from the center of the upper right sphere to the right. The indicators are consistent in the whole study.

According to ANSI/ADA No. 132, this study designed three reference models. The theoretical values of the crown model radial of the top surface and height were set to 3.5 mm and 6.0 mm, respectively, the radial of the top surface and height of the inlay model were set to 4.0 and 6.0 mm, respectively, and the center distance of a sphere with a long-distance specimen model diameter of 8.0 mm is set as R1 = 35.0 mm, R2 = 59.5 mm, R3 = 55.0 mm, R4 = 59.5 mm, R5 = 40.0 mm, and R6 = 40.0 mm. The 3D views and optical images are shown in Figure 1. Models of the samples were first drawn using CAD software (AutoCAD 2018, Autodesk, USA) and then exported in standard tessellation language (STL) format for computer numerical control milling. Samples were fabricated from stainless steel according to the STL file and washed three times in an ultrasonic bath at 30°C for 5 min each time. Finally, all the samples were sandblasted with a powder size of 80 *μ*m.

### 2.2. 3D Scanner

The oral scanners used in this experiment were an intraoral scanner (TRIOS SERIES3, 3Shape, Denmark) and two extraoral scanners (E4, 3Shape, Denmark & SHINING DS100+, Shining, China). The SHINING DS100+, whose field of view is 100 mm × 100 mm × 75 mm, uses blue light and point cloud to capture and form the corresponding image. Similarly, the other extraoral scanner, E4, has the same light source and imaging type as SHINING DS 100+. By contrast, the light source of the intraoral scanner was white light, and the STL file was obtained by splicing the image. More details regarding these scanners are presented in Table 1. Because it is unlikely that the fabricated samples exactly match the software plotted results, CMM was used in this study to measure the relevant indexes of the samples, and the results obtained were used as reference values.

### 2.3. Sample Scanning and Data Acquisition

**Figure 2:**
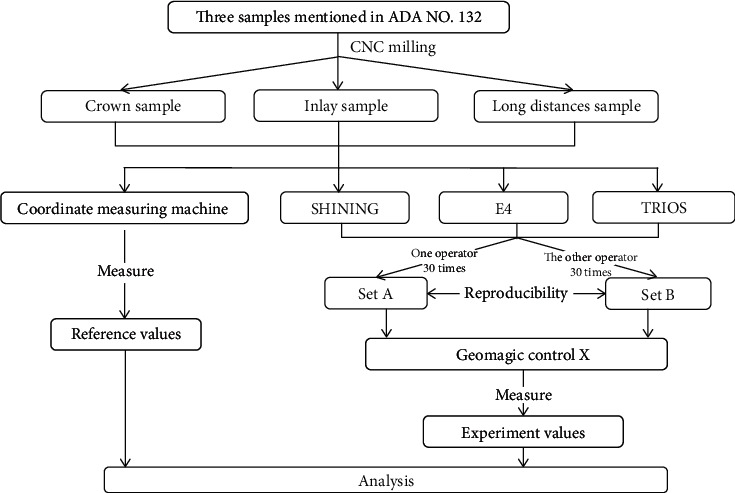
Specific processes of sample scanning and data acquisition based on repeatability and reproducibility experiments.

The fully trained operator performed 30 scans at 30 s intervals in strict accordance with the instructions for use under the same conditions to obtain a set named A (*N* = 30). The other operator performed 30 scans in a different environment to obtain a set named B (*N* = 30). Reverse engineering software (Geomagic Control X 2018; 3D SYSTEMS, USA) was used to measure a variety of indexes to complete the experiments (Figure 2).

Indexes marked in Figure 1 were measured, and then, data were compared and analyzed.

Using the principle of outlier elimination, if the difference between the data and the average value exceeds 1.96, standard deviations (i.e., outlier data) and the data are eliminated, and the scan and measurement are performed again. If there are more than two outliers, the experimental results will be canceled, and the experiment will be executed again.

### 2.4. Calculation of Trueness and Precision

Trueness and precision were quantified in terms of the relative error. ∆*d*_*M*_ represents the trueness of the test results. The smaller the value of ∆*d*_*M*_, the higher the trueness of the scanner. ∆*S*(*d*_*M*_) represents the precision of the test results. The smaller the ∆*S*(*d*_*M*_), the higher is the precision of the scanner.

Calculate the relative error according to Equations (1) and (2). [30].

(1) $$\begin{array}{c} \Delta dM=\left|\frac{d_{R}-d_{M}}{d_{R}}\right|. \end{array}$$

*d*_*R*_ represents the reference value of the sample, and *d*_*M*_ represents the measured value (including length, depth, height, and distance from the center).

(2) $$\begin{array}{c} \Delta S\left(dM\right)=\left|\frac{S}{d_{R}}\right|. \end{array}$$

*S* represents the standard deviation and *d*_*R*_ represents the reference value of the sample.

The ANSI/ADA No. 132 professional standard considers the relative error of the indexes in samples 1 and 2. Less than 0.01 mm is the acceptable range in the dental requirements category, and a relative error of less than 0.0025 mm represents the distance between sphere reference points to ensure that the acceptable threshold value in the dental requirements category is used as the threshold value.

### 2.5. Reproducibility Analysis

The reproducibility is reflected by comparing the values of indexes measured from sets A and B. The closer the results of 30 scans of experimental group B to that of experimental group A, the greater the reproducibility.

### 2.6. Statistical Analysis

Each set of data used the Shapiro-Wilk test for normality, which uses SPSS v.24.0 (IBM, USA) to determine the correlation between the scans from the different scanners in the experiment. The nonparametric Kruskal-Wallis test was used to analyze the differences in parameters. We then performed an independent samples *t*-test to analyze the reproducibility of each scan for different models.

### 2.7. Iterative Closest Point Matching Experiments

The values calculated from the scan images of the experiment were used to chart the accuracy and repeatability of the oral scanner. However, in addition to the overall detection indexes of the scanning image, the scanning effect of different scanners for the same part of the sample is also different, which cannot be reflected by the detection indexes. Therefore, it is necessary to perform matching experiments to intuitively compare the scanning differences among the three scanners for the same sample.

Each scanned STL file from one scanner was imported into Geomagic Control X to perform iterative closest point matching experiments with corresponding STL file obtained with another scanner. Before the matching experiments, excess parts of the STL file were removed for better results. Thereafter, the initial alignment is carried out, and then, the best-fit alignment between scan data is obtained by the least-squares method. Finally, 3D comparisons were conducted to obtain color images for the visual observation of the difference between the scanners. The average deviation of all points on the surface between two scanners scans is also calculated.

Because the scanned results were well distributed, the results of the iterative closest point matching experiments were also similar. A representative image was selected to represent the experimental results.

## 3. Results

### 3.1. Trueness

**Figure 3:**
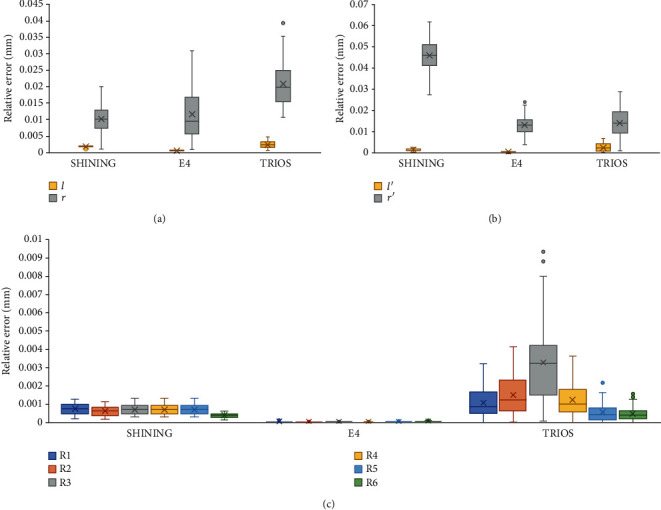
Boxplots of absolute mean trueness of crown, inlay sample, and long distance sample. Plot for the comparisons of (a) the crown, (b) the inlay, and (c) the long distance sample between SHINING, E4, and TRIOS.

**Figure 4:**
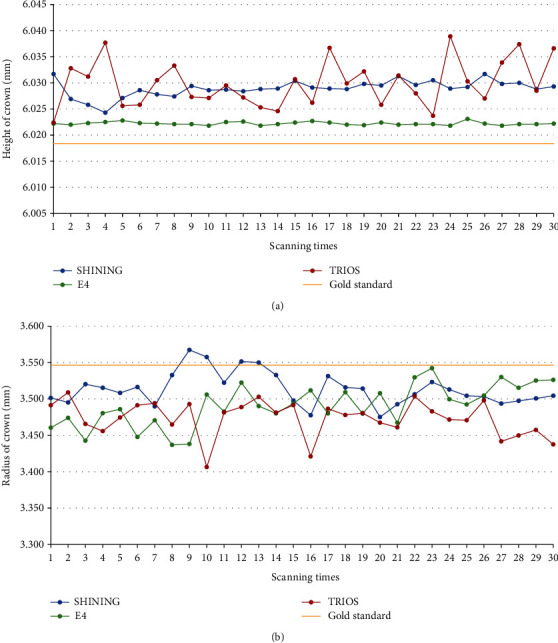
Plots for the comparisons of indexes between SHINING, E4, and TRIOS based on the results of the repeated measures. Plot for the comparisons of (a) the height of crown and (b) the radius of crown between SHINING, E4, and TRIOS.

**Figure 5:**
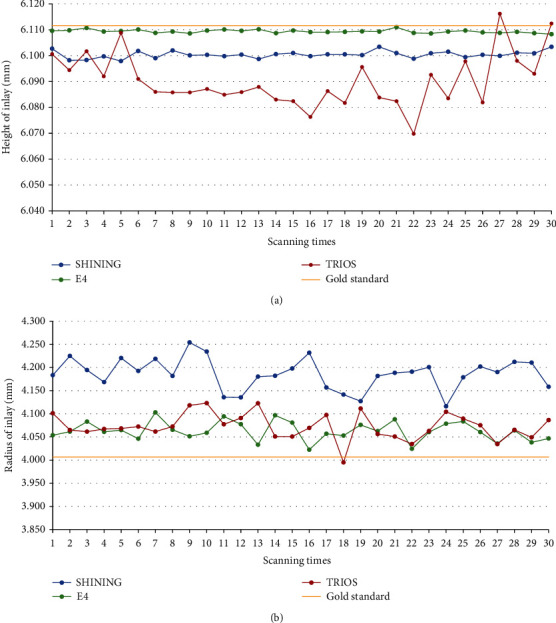
Plots for the comparisons of indexes between SHINING, E4, and TRIOS based on the results of the repeated measures. Plot for the comparisons of (a) the height of inlay and (b) the radius of inlay between SHINING, E4, and TRIOS.

**Figure 6:**
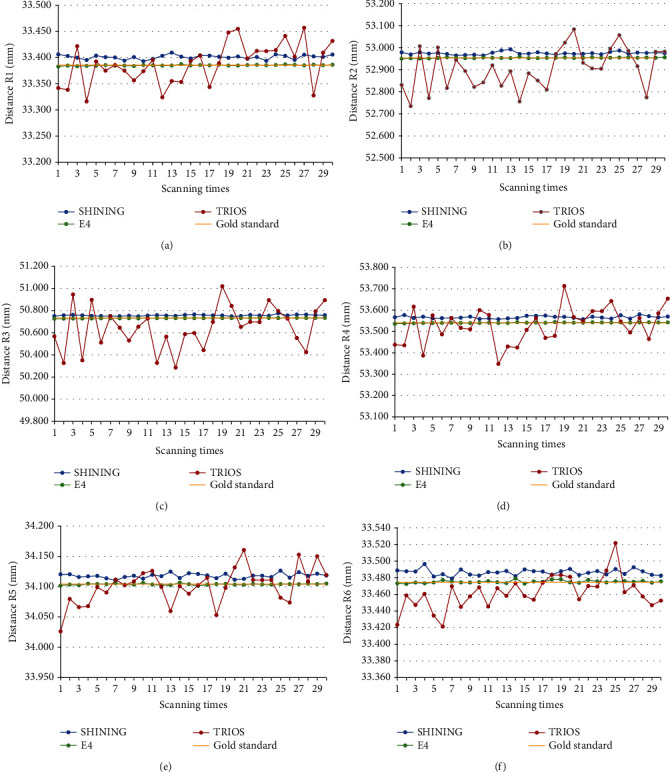
Plots for the comparisons of indexes between SHINING, E4, and TRIOS based on the results of the repeated measures. Plot for the comparisons of distance (a) R1, (b) R2, (c) R3, (d) R4, (e) R5, and (f) R6 between the datum points of spheres between SHINING, E4, and TRIOS.

The results of scanning the indexes of each sample with two extraoral scanners and an intraoral scanner are shown in Table 2, and the relative errors (∆*d*_*M*_) of the samples were compared with the ANSI/ADA No. 132 specified value.  Figure 3 is a box plot of trueness values. Figures 4–6 show the measurements and reference values for sample 1 (crown), sample 2 (inlay), and sample 3 scanned by SHINING, E4, and TRIOS scanners.

TRIOS had a large deviation in measuring R3, which exceeded the acceptable range in the dental requirement category. For sample 2, the SHINING scans showed larger relative errors than E4 and TRIOS, while for sample 1, the relative errors for E4 and TRIOS were larger than those for SHINING. Although the scanning data deviated from the reference value, the deviation was controlled between 0.001 and 0.184 mm. The E4 scan value was closest to the reference value.

There were significant differences in SHINING, E4, and TRIOS. The statistical differences between the three scanners are summarized in Table 3.

### 3.2. Precision

The results of scanning the indexes of each sample with two extraoral scanners and an intraoral scanner are shown in Table 4, and the relative errors (∆*S*(*d*_*M*_)) of the samples were compared with the ANSI/ADA No. 132 specified value. The results of SHINING, E4, and TRIOS scanning crowns and inlays were accepted by ANSI/ADA No. 132. The ∆*S*(*d*_*M*_) of E4 was the lowest of all three samples, showing the highest precision. SHINING, E4, and TRIOS have a large deviation from the reference value when scanning the inlay radius. The difference between repeated measurements of TRIOS is the largest, especially the distance between the datum points of the spheres.

### 3.3. Repeatability and Reproducibility of Samples

Regarding repeatability and reproducibility, most of the results tested by the two operators were statistically different (Table 5).

In the SHINING group, there was no statistically significant difference between groups A and B in the radius of the repeatedly scanned crowns and inlays. In the E4 group, there was no statistically significant difference between groups A and B in the radius of the scanned inlays. In the Trios group, there was no statistically significant difference between groups A and B in the radius of the repeatedly scanned crowns.

### 3.4. Iterative Closest Point Matching of Samples

The color bar represents qualitative information analysis. These differences between scanners are shown in the color bar [32]. While the color bar depicts deviations between -1 and 1 mm, acceptable errors between -0.01 and 0.01 mm are marked in green, better reflecting the differences between two scanners while scanning the same sample. A positive difference from yellow to red indicates a higher deviation from the reference model, and a negative difference from azure to dark blue indicates a lower deviation from the reference model.

**Figure 7:**
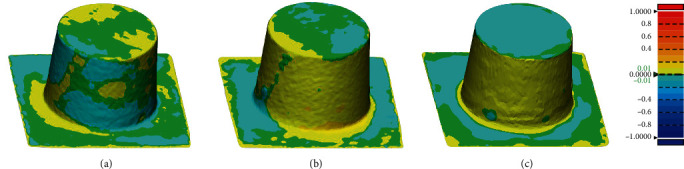
Selected representative crown scanned by SHINING, E4, and TRIOS for three-dimensional compare analysis. (a) E4 compares with TRIOS. (b) E4 compares with SHINING. (c) TRIOS compares with SHINING. ^∗∗∗^Color bar depicting deviations with settings at nominal between -0.01 mm and 0.01 mm and critical between -1 and 1 mm.

**Figure 8:**
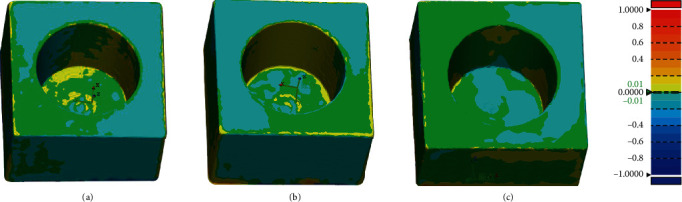
Selected representative inlay scanned by SHINING, E4, and TRIOS for three-dimensional compare analysis. (a) E4 compares with TRIOS. (b) E4 compares with SHINING. (c) TRIOS compares with SHINING. ^∗∗∗^Color bar depicting deviations with settings at nominal between -0.01 mm and 0.01 mm and critical between -1 and 1 mm.

Figures 7 and 8 show that the local deviation between the two scanners occurs on the curved surface and deep areas.  Table 6 shows the RMS values between the two scanners, and the RMS values between the scanners is within 1.0 mm.

## 4. Discussion

The oral scanner is evaluated based on the “trueness” and “precision” specified in ISO 5725-1 [33], where trueness is defined as the consistency between the test result and the acceptable reference value. Precision is defined as the closeness of the independent test results obtained under the specified conditions. The accuracy of the scanner was verified by a combined evaluation of trueness and precision.

This study is aimed at evaluating the trueness and precision of three oral scanners based on three samples provided by ANSI/ADA No. 132. The CMM was used to measure the data obtained from the original sample as a reference value, and the results were compared with those of the three scanners. Experiments were conducted to further analyze the accuracy and reproducibility of the scanners [28, 34–38]. At the same time, some pits generated in the scanning process are introduced into the interactive closest point matching to reflect the differences among the three scanners.

The calculated ∆*d*_*M*_ and ∆*S*(*d*_*M*_) indicate that although there were a few large numerical errors (e.g., the average value of the relative error of TRIOS for the radius of sample 1 is 0.0260 mm, and the average value of the relative error of SHINING for the radius of sample 2 is 0.0448), most of the trueness and precision were acceptable, and the accuracy was within the acceptable range. The accuracy of E4 was better than that of SHINING and TRIOS. In terms of reproducibility, most of the results tested by the two operators were statistically different.

As in previous studies [28, 34–39], the CMM measurement sample was used to obtain more accurate standard values. It should be noted that there is always an error between the results of the mechanical scan and the actual values of the sample. However, the CMM used in this study has high accuracy, and using CMM measurement samples as the reference value is also allowed in ANSI/ADA No. 132 [20, 40–42]. Inevitably, many errors were still present during the experiment.

The results of the repeated measures of TRIOS have a large fluctuation range. The high relative error of TRIOS may be related to the image acquisition method of the scanner and the operation of the operator. The extraoral scanner can construct the shape of the object effectively based on the point cloud obtained in the 3D point coordinate system, while the intraoral scanner uses the best-fitting algorithm to stitch the scanned images together. When the surface shape of the scanned object is complex, image alignment is easier, but when the scanned object is flat and smooth (such as edentulous jaws), images are more prone to errors, causing distortion of the STL file [43]. The farther away from the start scanning point and the greater the splicing times, the lower the accuracy of the data. Therefore, some studies suggested placing complex geometry near the edentulous area to improve scanning accuracy when using an intraoral scanner [43–45]. At the same time, previous studies have shown that the posterior dental arch exhibits greater errors during intraoral scanning [10, 44, 46]. Moreover, the handheld intraoral scanner oscillates during scanning and needs to constantly change the coordinates [26]. Therefore, each time the image of the scanner is stitched, the processing and fitting errors increase, resulting in inaccurate measurement and image distortion [47]. Therefore, as the complexity and geometry of tooth preparation increases, intraoral scans become inaccurate [25, 38, 39, 48]. Michaeli et al. suggested alleviating the experimental error by increasing the scanning angle and scanning times [49]. The extraoral scanner automatically scans the fixed model at different angles to reduce the influence of the operator on the scanning process. Therefore, the operator's impact on the extraoral scanner is much less than that on the intraoral scanner [38]. However, the two extraoral scanners and the intraoral scanner in this study showed poor reproducibility. The authors speculate that this may be related to scanning in different environments.

The best match alignments between scanners were conducted using the best-fit algorithm of Geomagic Control X. Each point in the source point cloud was best aligned with the closest point in the reference point cloud. The offset between digital models obtained from every two scanners was visualized by color mapping. Geomagic Control X can efficiently detect the differences between irregular shapes, which is especially suitable for analyzing oral models. However, because the scanning offset reflected by Geomagic Control X is not applicable to the evaluation of sphere spacing, iterative closest point matching is not considered for the sphere sample in this study.

In Tables 2 and 4, the relative errors of E4, SHINING, and TRIOS in the scan model radius exceeded the acceptable range in the dental requirement category. In Figure 7, scanners showed relatively large differences when scanning curved surfaces and deep area. This may be caused by the energy lost by the light source because of the reflection when the surface of the scanned sample moves, which affects scanned by the scanner, and the curved surface is more likely to cause light reflection [50]. In Figure 8, comparing between each other, three scanners showed obvious deviation when scanning the bottom of the inlay, which may also be related to the fact that the scanner light source cannot perform detailed scanning at the bottom. Different light sources also affect the accuracy of the scanner [10, 51]. Araki et al. scanned under different light sources and evaluated the results. They found that the most suitable lighting conditions for digital impression scanning were 3900 K and 500 lux [52]. However, in the iterative closest point matching experiments, the deviations produced by the best matching process are all less than 0.01 mm.

In previous studies, different methods have been used to evaluate the accuracy of diverse oral scanners. Intraoral scanner systems were found to be less accurate than extraoral scanners, and the ability of extraoral scanners to scan the edges of the crown is also better than that of intraoral scanners [25, 39]. This is consistent with the results of our experiments. Cai et al. evaluated the accuracy of SHINING, CEREC, and TRIOS by scanning an international standard sphere model, and the results showed that the accuracy of the intraoral scanners was better than that of the extraoral scanner [53]. However, in this study, the accuracy of the extraoral scanner E4 was significantly better than that of the intraoral scanner TRIOS.

This study has some limitations, as it was conducted in vitro and did not simulate actual clinical conditions. The effects of temperature and humidity of the oral environment, saliva and blood, soft tissues, patient movement, oral cavity, and scanning laser angle of incidence were not considered [10, 54, 55]. At the same time, many items in the oral cavity, such as implants (ceramics, metals, and composite resins), dentin, enamel, oral soft tissue, and different materials and geometric shapes, also affect the accuracy of scanning. The scanned samples presented in this study have different light effects from the soft tissue around the oral cavity [26, 56]; therefore, there are some limitations. In future studies, we will simulate a clinical setting and increase the number of scanning groups to further evaluate the accuracy of different scanners.

## 5. Conclusion

This study evaluated the accuracy of the three scanners by measuring ANSI/ADA No. 132 provided reference models.

1. There was significant difference between the scanning results of the three scanners, with E4 showing the best reproducibility
2. Whether scanning crown, inlay, or arch, the extraoral scanner showed fewer distortions than the intraoral scanner, with E4 showing the least error
3. Most of the scanning errors of the extraoral and intraoral scanners were within the acceptable range of the ADA standard. However, the effect of scanning the sample radius is poor
4. Relatively large differences between scanners occurred when scanning curved surfaces and the deep areas of the inlay

## Figures and Tables

**Table 1:** Product information of three oral scanners (SHINING, TROIS, E4) and coordinate measuring machine (CMM, NC8107).

Scanner	Manufacturer	Light source	Powder	Metal reflection	Output format	Imaging type
SHINING DS100+	Shining	Blue light	Free	Diffuse reflection	Proprietary or STL	Video (point cloud data)
TRIOS SERIES3	3Shape	White light	Free	Diffuse reflection	Proprietary or STL	Image
E4	3Shape	Blue light	Free	Diffuse reflection	Proprietary or STL	Video (point cloud data)
NC8107	Leader metrology	—	—	—	—	—

**Table 2:** Test protocol template for relative error ∆*d*_*M*_ of crown, inlay sample, and long distance sample using three scanners (SHINING, E4, TROIS).

Test object	∆*d*_*M*_ (*μ*m)		
	SHINING	E4	TRIOS
Crown sample			
*l*	1.75 ± 0.25	8.10 ± 0.05	1.91 ± 0.73
*r*	9.96 ± 4.88	13.99 ± 7.96	20.60 ± 6.73
Inlay sample			
*l*′	1.82 ± 0.22	0.10 ± 0.36	3.54 ± 1.52
*r*′	44.83 ± 8.19	13.99 ± 5.09	16.70 ± 6.51
Long distance sample			
R1	0.46 ± 0.12	0.02 ± 0.02	0.97 ± 0.64
R2	0.39 ± 0.12	0.02 ± 0.02	1.63 ± 1.10
R3	0.48 ± 0.12	0.03 + 0.03	3.31 ± 2.46
R4	0.47 ± 0.11	0.02 ± 0.02	1.26 ± 0.89
R5	0.40 ± 0.11	0.03 ± 0.02	0.64 ± 0.57
R6	0.35 ± 0.11	0.04 ± 0.03	0.55 ± 0.43

**Table 3:** Analysis of the difference in the SHINING, E4, and TROIS.

Test object	*P* value		
	SHINING-E4	SHINING-TRIOS	E4-TRIOS
Crown sample			
*l*	<.01^∗∗^	.06	<.01^∗∗^
*r*	1.00	<.01^∗∗^	<.01^∗∗^
Inlay sample			
*l*′	<.01^∗∗^	1.00	<.01^∗∗^
*r*′	<.01^∗∗^	<.01^∗∗^	1.00
Long distance sample			
R1	<.01^∗∗^	.04	<.01^∗∗^
R2	<.01^∗∗^	<.01^∗∗^	1.00
R3	<.01^∗∗^	<.01^∗∗^	1.00
R4	<.01^∗∗^	<.01^∗∗^	<.01^∗∗^
R5	<.01^∗∗^	<.01^∗∗^	.17
R6	<.01^∗∗^	<.01^∗∗^	.35

^∗^ indicates a difference at the significance level of 0.05; ^∗∗^ indicates a difference at the significance level of 0.01. The normal distribution test (Shapiro-Wilk test) and an independent-samples Kruskal-Wallis test.

**Table 4:** Test protocol template for relative error ∆*S*(*d*_*M*_) of crown, inlay sample, and long distance sample using three scanners (SHINING, E4, and TROIS).

Test object	∆*S*(*d*_*M*_) (*μ*m)		
	SHINING	E4	TRIOS
Crown sample			
*l*	0.26	0.10	0.73
*r*	6.21	7.96	6.74
Inlay sample			
*l*′	0.22	0.10	1.66
*r*′	0.82	5.09	6.99
Long distance sample			
R1	0.12	0.27	1.16
R2	0.12	0.03	1.76
R3	0.12	0.04	3.77^∗∗^
R4	0.11	0.03	1.53
R5	0.11	0.03	0.85
R6	0.11	0.05	0.58

^∗^Greater than 10 *μ*m; ^∗∗^greater than 2.5 *μ*m.

**Table 5:** Repeatability and reproducibility in the SHINING, E4, and TROIS.

Test object	SHINING			E4			TRIOS		
	Mean ± SD		*P*	Mean ± SD		*P*	Mean ± SD		*P*
	A (mm)	B (mm)		A (mm)	B (mm)		A (mm)	B (mm)	
Crown sample									
*L*	6.03 ± 0.00	6.03 ± 0.00	.027^∗^	6.02 ± 0.00	6.02 ± 0.00	.000^∗∗^	6.03 ± 0.00	6.04 ± 0.01	.000^∗∗^
*R*	3.51 ± 0.02	3.51 ± 0.01	.231	3.49 ± 0.03	3.52 ± 0.02	.000^∗∗^	3.47 ± 0.02	3.47 ± 0.02	.724
Inlay sample									
*l*′	6.10 ± 0.00	6.10 ± 0.00	.000^∗∗^	6.11 ± 0.00	6.11 ± 0.00	.000^∗∗^	6.09 ± 0.01	6.10 ± 0.01	.000^∗∗^
*r*′	4.19 ± 0.03	4.20 ± 0.03	.237	4.06 ± 0.02	4.06 ± 0.01	.109	4.07 ± 0.03	4.06 ± 0.03	.017^∗^
Long distance sample									
R1	33.40 ± 0.00	33.42 ± 0.00	.000^∗∗^	33.39 ± 0.00	33.39 ± 0.00	.003^∗∗^	33.39 ± 0.04	33.42 ± 0.03	.002^∗∗^
R2	52.98 ± 0.01	53.00 ± 0.01	.000^∗∗^	52.95 ± 0.00	52.96 ± 0.00	.017^∗^	52.90 ± 0.09	52.95 ± 0.09	.037^∗^
R3	50.76 ± 0.01	50.78 ± 0.01	.000^∗∗^	50.73 ± 0.00	50.73 ± 0.00	.000 ^∗∗^	50.65 ± 0.19	50.71 ± 0.19	.231
R4	53.57 ± 0.01	53.58 ± 0.01	.000^∗∗^	53.54 ± 0.00	53.54 ± 0.00	.051	53.53 ± 0.08	53.56 ± 0.08	.138
R5	34.12 ± 0.00	34.12 ± 0.00	.000^∗∗^	34.10 ± 0.00	34.10 ± 0.00	.015^∗^	34.10 ± 0.03	34.11 ± 0.02	.159
R6	33.49 ± 0.00	33.49 ± 0.00	.006^∗∗^	33.48 ± 0.00	33.47 ± 0.00	.012^∗^	33.46 ± 0.02	33.47 ± 0.02	.022^∗^

^∗^ indicates a difference at the significance level of 0.05; ^∗∗^ indicates a difference at the significance level of 0.01. The normal distribution test (Shapiro-Wilk test) and an independent-samples *t*-test.

**Table 6:** RMS values for the 3D-fitting results.

Test group	RMS	
	Crown (mm)	Inlay (mm)
E4-TRIOS	0.0559	0.0669
E4-SHINING	0.0592	0.044
TRIOS-SHINING	0.0408	0.1345

## Data Availability

The data used to support the findings of this study are included in this article.
